# Quantitative phosphoproteomic analysis provides insights into the aluminum-responsiveness of Tamba black soybean

**DOI:** 10.1371/journal.pone.0237845

**Published:** 2020-08-19

**Authors:** Rongrong Han, Yunmin Wei, Yonghong Xie, Lusheng Liu, Caode Jiang, Yongxiong Yu

**Affiliations:** College of Animal Science and Technology, Southwest University, Beibei, Chongqing, China; Pacific Northwest National Laboratory, UNITED STATES

## Abstract

Aluminum (Al^3+^) toxicity is one of the most important limitations to agricultural production worldwide. The overall response of plants to Al^3+^ stress has been documented, but the contribution of protein phosphorylation to Al^3+^ detoxicity and tolerance in plants is unclear. Using a combination of tandem mass tag (TMT) labeling, immobilized metal affinity chromatography (IMAC) enrichment and liquid chromatography-tandem mass spectrometry (LC-MS/MS), Al^3+^-induced phosphoproteomic changes in roots of Tamba black soybean (TBS) were investigated in this study. The Data collected in this study are available via ProteomeXchange with the identifier PXD019807. After the Al^3+^ treatment, 189 proteins harboring 278 phosphosites were significantly changed (fold change > 1.2 or < 0.83, *p* < 0.05), with 88 upregulated, 96 downregulated and 5 up-/downregulated. Enrichment and protein interaction analyses revealed that differentially phosphorylated proteins (DPPs) under the Al^3+^ treatment were mainly related to G-protein-mediated signaling, transcription and translation, transporters and carbohydrate metabolism. Particularly, DPPs associated with root growth inhibition or citric acid synthesis were identified. The results of this study provide novel insights into the molecular mechanisms of TBS post-translational modifications in response to Al^3+^ stress.

## Introduction

Aluminum (Al^3+^) stress poses a major constraint for plant productivity in acidic soils, which constitute approximately 50% of arable lands worldwide [1]. At pH < 5.0, soluble aluminum, which occurs mainly in the forms of Al^3+^ and Al(OH)^2+^, damages nuclei, reduces mitotic activity, inhibits root elongation and suppresses the absorption of water and nutrients [2–4]. The mechanisms underlying plant resistance to Al^3+^ have been the focus of recent research. An in-depth understanding of Al^3+^-resistance mechanisms will favor the development of cultivated species suitable for acidic soils.

Plants in acidic soil have developed Al^3+^ exclusion and tolerance mechanisms. The exclusion mechanism prevents Al^3+^ from entering root cells via immobilizing Al^3+^ in the cell wall or by forming stable nonphytotoxic chelates with organic acid anions (OAs), such as citrate, malate and oxalate, at the root apex [5, 6]. Internal tolerance mechanisms enable root cells to sequester Al^3+^ in vacuoles [7]. For decades, a number of proteins contributing to Al^3+^ tolerance have been identified in plants, and many studies have provided direct evidence linking Al^3+^-induced OA exudation from plant roots to malate and citrate efflux transporters (ALMTs), multidrug and toxic compound extrusion proteins (MATEs) and H^+^-ATPase activity in the plasma membrane [1, 8]. In *Stylosanthes* roots, our recent data revealed that the signaling cascades of Al^3+^-induced citrate exudation comprise heterotrimeric G-proteins, phosphoinositide phospholipase C (PLC), inositol triphosphate (IP_3_), diacylglycerol (DAG), Ca^2+^ and protein kinases [9].

Protein phosphorylation, which is one of the most important post-translational protein modifications, can modulate the functions of proteins. Salicylic acid (SA) can mitigate Al^3+^ toxicity by affecting a signaling pathway associated with protein phosphorylation in *Coffea arabica* L. suspension cells [10]. Importantly, OA transport proteins in response to Al^3+^ are post-translationally regulated by protein phosphorylation. In wheat (*Triticum aestivum*), Al^3+^-induced malate efflux as effectively blocked by K-252a, which is a broad range inhibitor of protein kinases [8]. Plasma membrane H^+^-ATPase was hyperphosphorylated after Al^3+^ treatment at a concentration below 50 μM in soybean (*Glycine max*) roots [11]. Our previous research also demonstrated that phosphorylation is responsible for the interaction of H^+^-ATPase and 14-3-3 protein, which leads to Al^3+^-stimulated citrate exudation in roots of Tamba black soybean (*Glycine max* cv. Tamba, TBS) [12–14]. Additionally, genome-wide association analysis [15], transcriptomics [9, 16] and proteomics [17] have allowed the exploration of the mechanisms of Al^3+^ resistance globally. However, phosphorylated proteins related to Al^3+^-induced citrate exudation at the global scale have rarely been reported.

In recent years, quantitative phosphoproteomics using tandem mass tag (TMT) labeling has provided useful information for subsequent functional studies, and has been applied to examinations of plant phosphorylated proteins during abiotic and biotic stress [18, 19] and during plant growth and development [20]. Hence, this approach was employed to evaluate differentially regulated phosphoproteins induced by Al^3+^ stress in roots of the Al^3+^-resistant cultivar TBS. The identified Al^3+^-induced phosphoproteins can enable further investigation of the Al^3+^ tolerance mechanisms related to citrate secretion from roots of TBS plants.

## Materials and methods

### Cultivation of TBS plants

TBS seeds were disinfected with 1% sodium hypochlorite for 20 min and washed in double-distilled water three times. The seeds were incubated on moistened filter papers at 25°C. After germination, the seedlings were transplanted into 8 L aquariums containing 1/2 Hoagland’s nutrient solution (pH 6.0), which was renewed every two days. The seedlings were grown in an artificially lit room at 27°C/22°C (day/night) with 14 h of light (200 μmol/m^2^/s) for 2 weeks.

### Al^3+^ treatment and measurement of the relative root growth (RRG), citrate content and citrate secretion

For treatment, uniform seedlings were pre-grown overnight in a 0.5 mM CaCl_2_ solution (pH 4.5) at 25°C under constant light as described above. Then, every ten seedlings were transferred into solutions containing 0 (control) or 50 μM AlCl_3_ (both containing 0.5 mM CaCl_2_, pH = 4.5) for 72 h according to previous studies with minor modifications [21]. The harvested root tips were stored in a −80°C freezer after snap-freezing in liquid nitrogen for future use. For phosphoproteomic analysis, there were three replicates per treatment, namely, 0 μM (1), 0 μM (2), and 0 μM (3) for the control groups, and 50 μM (1), 50 μM (2), and 50 μM (3) for the Al^3+^-treatment groups.

For the measurement of RRG, TBS plants were transferred into a 0 (control) or 50 μM AlCl_3_ solution (both containing 0.5 mM CaCl_2_, pH = 4.5) for 24, 48 and 72 h, respectively. Root length was measured before and after treatment. RRG analysis was carried out according to the procedures described by Min [13]. The analysis was performed with three replicates with 5 plants per replicate.

For the measurement of the citrate content and citrate secretion, TBS plants were transferred into 0 and 50 μM AlCl_3_ solutions (containing 0.5 mM CaCl_2_, pH = 4.5), and the roots and root exudates were collected at 3, 6, 12, 24, 48 and 72 h. Then, 0.1 g root tips was ground in 1 mL ddH_2_O and centrifuged at 12,000 rpm for supernatant collection. The citrate content was measured by enzymic determination according to Zhao [22]. There were three replicates for each time point with 5 plants per replicate.

### Protein extraction, digestion, TMT labeling and phosphopeptide enrichment

Proteins extraction was carried out according to the procedures described by Sun [18] with slight modification. The protein concentration was evaluated with the BCA kit (Beyotime, Shanghai, China). For protein digestion, trypsin (Promega, Wisconsin, USA) was added at a 1:50 trypsin-to-protein mass ratio for overnight digestion and at a 1:100 trypsin-to-protein mass ratio for another 4 h digestion. After the digested solutions were desalted with a Strata X C18 SPE column (Phenomenex, California, USA) and vacuum-dried, they were dissolved in 0.5 M TEAB, and labeled as 0 μM (3)-126, 0 μM (2)-127, 0 μM (1)-128, 50 μM (3)-129, 50 μM (2)-130, and 50 μM (1)-131 using a TMT kit (Thermo Scientific, Waltham, USA). The TMT-labeled peptides were separated into 60 fractions with a gradient of 8 to 32% acetonitrile (pH 9.0) over 60 min. The collected peptides were recombined into 8 fractions and dried via vacuum centrifugation.

For phosphopeptides enrichment, the peptides in each fraction were incubated under gentle vibration in an immobilized metal affinity chromatography (IMAC) microsphere suspension in loading buffer (50% acetonitrile/6% trifluoroacetic acid). The phosphopeptides absorbed by the IMAC microspheres were collected by centrifugation and were washed with 50% acetonitrile plus 6% trifluoroacetic acid and with 30% acetonitrile plus 0.1% trifluoroacetic acid. The enriched phosphopeptides were eluted with 10% NH_4_OH and then lyophilized for liquid chromatography-tandem mass spectrometry (LC-MS/MS) analysis.

### LC-MS/MS and MS/MS data analysis

The peptides were dissolved in solvent A (0.1% formic acid) and separated with a gradient according to the following procedure, i.e., 6 to 23% solvent B (0.1% formic acid in 98% acetonitrile) over 26 min, 23 to 35% solvent B for 8 min, and 80% solvent B for 6 min. The tryptic peptides were separated using an EASY-nLC 1000 UPLC system at a constant flow rate of 400 nL/min and then subjected to ionization using an NSI source, followed by tandem mass spectrometry (MS/MS) analysis in Q Exactive^TM^ Plus (Thermo Scientific, Waltham, USA) online-coupled to the UPLC. The electrospray voltage applied was 2.0 kV. A full mass scan over the range of m/z 350 to 1800 was obtained with a resolution of 70000, while the fragments were detected in the Orbitrap at a resolution of 17,500 at m/z 100. For MS/MS, the normalized collision energy (NCE) was set to 28%, the dynamic exclusion time of the MS/MS scanning was set to 15 s, and the automatic gain control (AGC) was set at 5E4.

The Maxquant search engine (v.1.5.2.8) was applied to process the MS/MS data according to Chen’s work [23]. Tandem mass spectra of peptides were searched against the Phytozome 12.1-*Glycine max Wm82*.*a2*.*v1* database concatenated with the reverse decoy database. The false discovery rate (FDR) was adjusted to < 1% and the minimum score for modified peptides was > 40. The number of unique peptides was set to ≥2.

### Bioinformatics methods

Functional annotation of differentially phosphorylated proteins (DPPs) was performed using the Uniprot-GoA database (http://www.ebi.ac.uk/GOA/) for GO annotation. The Kyoto Encyclopedia of Genes and Genomes (KEGG) online service tools (https://www.kegg.jp/kegg/) were used to perform KEGG pathway mapping of the annotated protein KEGG database descriptions. Only categories with a two-tailed Fisher’s corrected *p*-value < 0.05 were considered to indicated a significant enrichment of DPPs against all identified proteins. The updated version of WOLFPSORT (http://www.genscript.com/wolf-psort.html) was used to predict subcellular localizations. Soft motif-X was applied to analyze the phosphorylation motifs at specific positions of modified-13-mers (6 amino acids upstream and downstream of the site) in the whole protein sequences. The significance threshold was set to *p* < 10^−6^, and the minimum occurrence of motifs was set to 20. Protein-protein interactions (PPIs) were analyzed by the Search Tool for the Retrieval of Interaction Genes/Proteins (STRING) database (http://string-db.org/). The interaction confidence score was set at a high level (≥ 0.7). The interaction network from STRING was visualized in Cytoscape (http://www.cytoscape.rog/). The thresholds used for the identification of significant DPPs were set at a fold change > 1.2 or < 0.83 and *p* < 0.05.

## Statistical analysis

Data for the root growth, citrate content and secretion are presented as the mean ± the standard error of the mean (SEM). One-way ANOVA followed by Duncan’s test was used to compare significance among treatments. Statistical significance was set to *p* < 0.05. SPSS Statistics19 and GraphPad (Version 8.3.0) were used for statistical analysis and graph preparation, respectively.

## Results

### Effect of the Al^3+^ treatment on the RRG, citrate content and secretion in TBS roots

**Fig 1:**
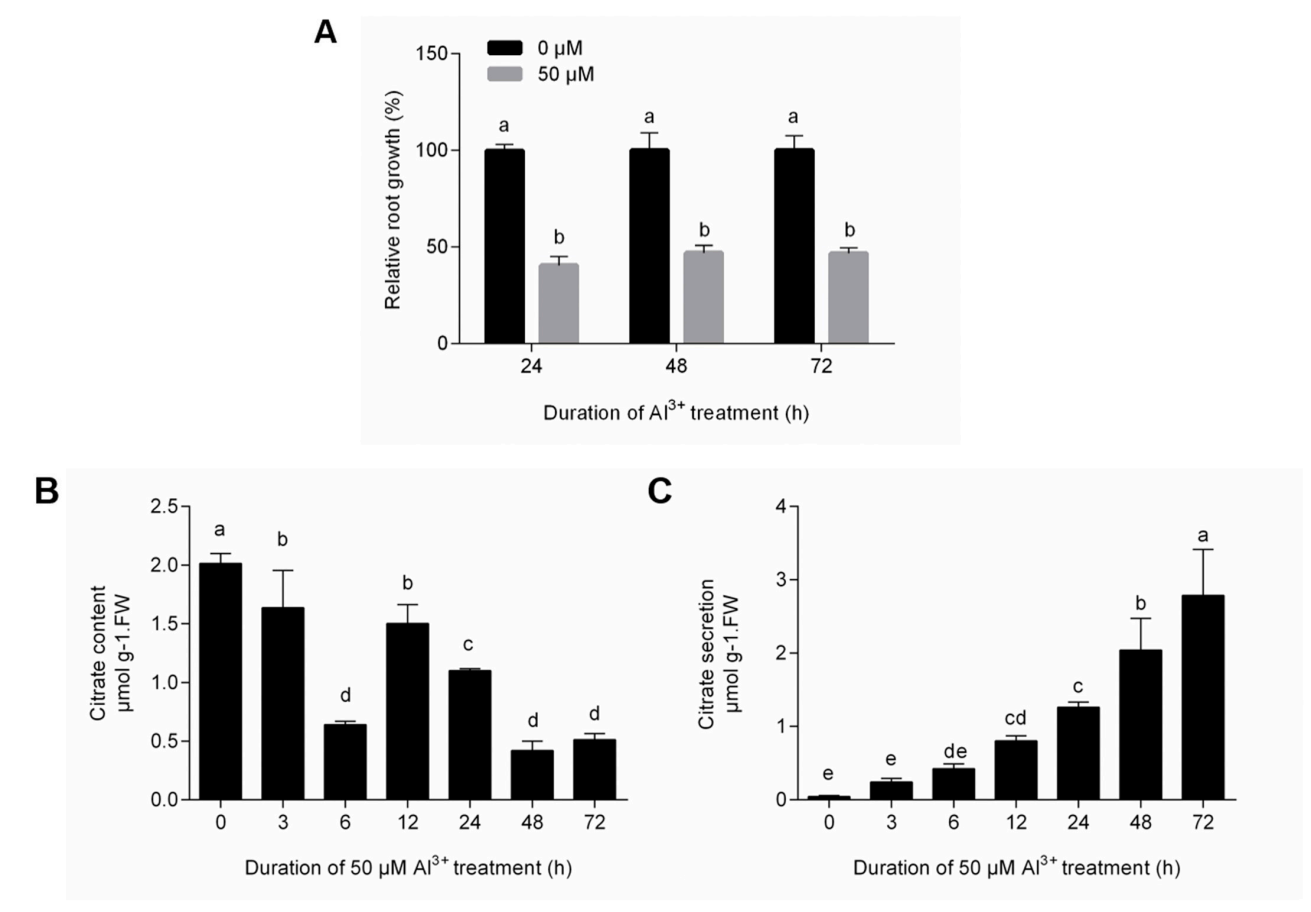
Effect of the Al^3+^ treatment on TBS roots. **(A)** Relative root growth; **(B)** Citrate content; **(C)** Citrate secretion. The bars are the means ± standard deviation of three replicates. Different letters above columns indicate significance (Duncan, *p* < 0.05).

Under Al^3+^ stress, root elongation of the plants was inhibited. The RRG of TBS was reduced by > 50% within 24, 48 and 72 h after the Al^3+^ treatment (*p* < 0.05) (Fig 1A). The Al^3+^ treatment significantly decreased the citrate content in the root tips within 3, 6, 12, 24, 48 and 72 h (*p* < 0.05) (Fig 1B), while citrate secretion from the root tips was significantly increased (*p* < 0.05) (Fig 1C). These results are similar to those of Eticha’s research [24].

### Analyses of phosphorylated proteins and sites in TBS roots under Al^3+^ stress

The phosphoproteomic profiles were evaluated in root tips of TBS seedlings after the Al^3+^ treatment for 72 h. The mass spectrometry proteomics data have been deposited to the ProteomeXchange Consortium via the PRIDE partner repository with the dataset identifier PXD019807 (Username: reviewer89780@ebi.ac.uk, Password: NKBxNBA3). In total, 6156 phosphopeptides representing 1415 proteins were obtained, among which 3245 phosphosites from 1280 proteins provided quantitative information (S1 Table). The peptide mass error was distributed mainly between -5 and 5 ppm (S1A Fig). Approximately 29.96% of the peptides were phosphorylated at a single site, while phosphopeptides with two, three or four sites constituted 28.48, 17.81 and 7.62% of the total, respectively (S1B Fig). Additionally, the lengths of more than 95.42% peptides were distributed between 7 and 21 amino acids (aa), consistent with the properties of tryptic peptides.

### DPPs in response to the Al^3+^ treatment

**Fig 2:**
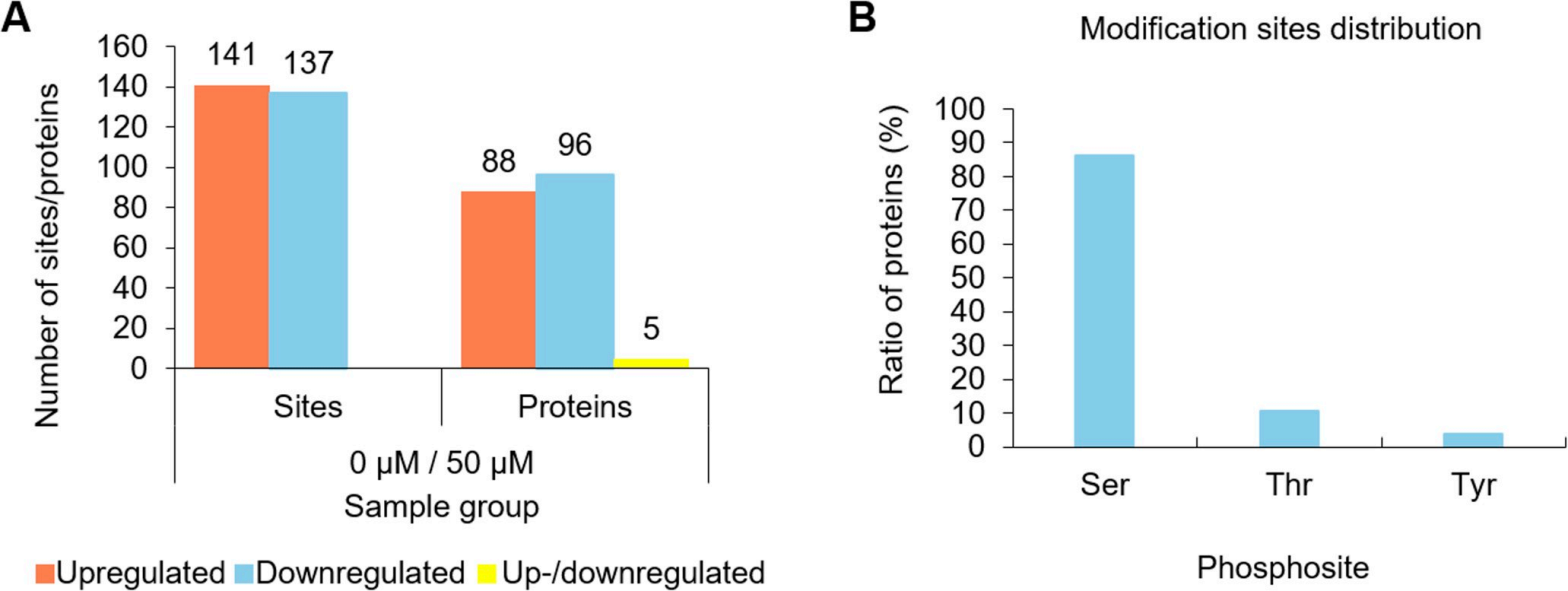
Distribution of phosphorylated proteins and phosphorylated sites. **(A)** The number of up-, down-, and up-/downregulated phosphorylated sites and proteins in the root of Al^3+^-treated (50 μM) and untreated (0 μM) plants. **(B)** Distribution of phosphorylation sites at serine, threonine and tyrosine residues. Three biological replicates were performed.

The DPPs in roots between the Al^3+^-treated (50 μM) and non-treated control groups (0 μM) were examined. A total of 189 phosphoproteins, including 88 upregulated in regard to phosphorylation (> 1.2-fold, *p* < 0.05), which possessed 135 phosphorylation sites, and 96 downregulated in regard to phosphorylation containing 131 phosphorylation sites (< 0.83-fold, *p* < 0.05), were differently phosphorylated under Al^3+^ stress (Fig 2A, S2 Table). In addition, 5 DPPs contained both upregulated (6) and downregulated (6) phosphorylation sites. Of all the phosphorylation sites in the DPPs, 85.97% were serine phosphorylation, and threonine and tyrosine phosphorylation represented 10.43 and 3.60%, respectively (Fig 2B, S2 Table).

### Functional classification of the DPPs

The DPPs were annotated using gene ontology (GO) terms based on biological processes (BP), cellular components (CC), and molecular functions (MF). For the BP category, the DPPs were classified into metabolic processes, cellular processes, single-organism processes, localization, cellular component organization or biogenesis, and other (S2A Fig). In the CC category, the DPPs were grouped into cell processes, organelle processes, macromolecular complex processes, and membrane processes (S2B Fig). Regarding the MF category, the DPPs belonged to the categories of binding, catalytic activity, molecular function regulator, transporter activity, and other (S2C Fig).

The subcellular localization of the DPPs was further analyzed. Most of the DPPs were located in the nucleus, chloroplast, cytoplasm and plasma membrane (S3A Fig). While 56% of the upregulated phosphoproteins were located in the nucleus, 19 and 13% were located in the chloroplast and cytoplasm, respectively (S3B Fig). Of the downregulated phosphoproteins, 66% were located in the nucleus, whereas 11, 9 and 8% were located in the chloroplast, cytoplasm, and plasma membrane, respectively (S3C Fig).

### Enrichment analysis of the DPPs under Al^3+^-treatment

GO enrichment analysis according to CC terms showed that the upregulated DPPs were over-represented in the cytoplasmic part, while the DPPs were downregulated in the membrane-bounded organelle (S4 Fig, S3 Table). In terms of the MF, the enriched terms in the upregulated DPPs were related to enzyme regulator activity, hydrolase activity (acting on glycosyl bonds), GTPase regulator activity, nucleoside-triphosphatase regulator activity, GTPase activator activity, enzyme activator activity, and O-acyltransferase activity, whereas the downregulated DPPs were associated with histone binding, RNA binding, signal transducer activity, translation initiation factor activity, and nucleic acid binding. The BP terms dominant for upregulated DPPs included the processes of glycerolipid metabolism, protein modification, cellular protein modification, and phosphorylation, but the downregulated DPPs were enriched in the processes of RNA metabolism and nucleic acid metabolism. These results indicated that the DPPs were related to stress, homeostasis, amino acid metabolism, transport processes and energy metabolic processes.

KEGG enrichment analysis revealed that all the DPPs were enriched in three categories, namely, spliceosome, mRNA surveillance and RNA transport (S4 Table). The upregulated DPPs were involved in RNA degradation and endocytosis pathways (S5A Fig, S4 Table). The downregulated DPPs were associated with spliceosome, RNA transport, and the mRNA surveillance pathway (S5B Fig, S4 Table). These results were consistent with the results of the GO terms, indicating that the DPPs were distributed to transport and RNA replication and modification.

The motifs were analyzed with the motif-X algorithm for the phosphorylation sites from the -6 to 6 positions in the DPPs. In all the phosphoproteins, 55 phosphorylation motifs were identified (8.71 ≤ motif score ≤ 43.05, S5 Table). The motifs distributed in the DPPs are listed in the S6 Table.

### PPI of phosphoproteins

**Fig 3:**
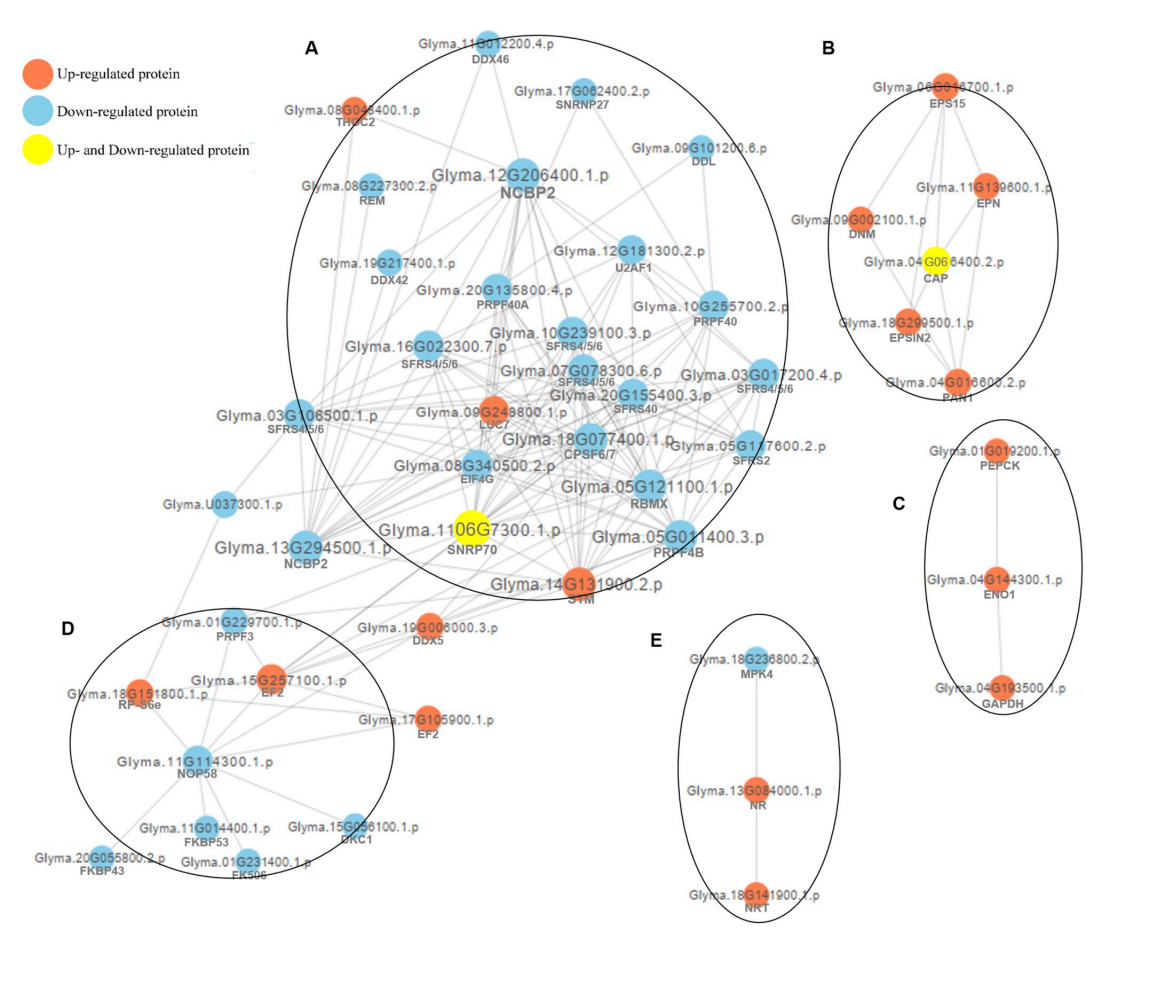
Protein-protein interaction networks of differentially phosphorylated proteins (DPPs) under Al^3+^ treatment in TBS seedlings. **(A)** Spliceosome; **(B)** Membrane trafficking; **(C)** Carbohydrate metabolism; **(D)** Ribosome biogenesis in eukaryotes; **(E)** Signal transduction.

Usually, proteins take part in diverse cellular processes via forming a complex regulatory network. To reveal the functional relationships among the DPPs, the PPI network was constructed using STRING version 10.0 and a confidence score of ≥ 0.7 (high confidence). As shown in Fig 3, the PPI network was involved in spliceosome (A), membrane trafficking (B), carbohydrate metabolism (C), ribosome biogenesis in eukaryotes (D), and signal transduction (E). The detailed information of the nodes is listed in S7 Table.

### DPPs involved in signal transduction

Seventeen DPPs involved in cell signaling were increased, while 5 DPPs were decreased in phosphorylation under the Al^3+^ treatment (Table 1, S2 and S8 Tables). These DPPs were associated with serine/threonine kinases (9), diacyglycerol and inositol metabolism (3), the ras family (4), kinase cascades (3) and others (3). Among serine/threonine kinases, BLUS1 (Glyma.10G173000.5.p, Glyma.20G217200.2.p), EDR1 (Glyma.12G128700.3.p), HT1 (Glyma.13G238400.7.p), and DDB (Glyma.15G261200.4.p) were observed to be hyperphosphorylated under Al^3+^ stress, while PRPF4B (Glyma.05G011400.3.p) and PBS1 (Glyma.13G289900.2.p) were hypophosphorylated. For diacyglycerol and inositol metabolism, 2 diacylglycerol acyltransferase (Glyma.07G036400.1.p and Glyma.09G065300.2.p) and 1 phosphatidylinositol 4-phosphate 5-kinase 7 (Glyma.08G186400.3.p) were determined to be upregulated in regard to phosphorylation. Additionally, 4 ADP-ribosylation factor GTPase-activating proteins (Glyma.08G039600.1.p, Glyma.08G271700.1.p, Glyma.09G188300.2.p and Glyma.19G172700.1.p) belonging to the ras family were all hyperphosphorylated by Al^3+^. Regarding kinase cascades, CPK1 (Glyma.10G120300.2.p) and CKI (Glyma.13G105900.2.p) were hyperphosphorylated, while MAPK (Glyma.18G236800.2.p) was hypophosphorylated.

**Table 1:** Summary of the phosphoproteins affected by Al^3+^.

Protein category	Total	Upregulated	Downregulated	Up-/downregulated
**Signal transduction**	22	17	5	0
**Transcription**	5	2	3	0
**Translation and post-translational modification**	15	6	8	1
**Carbohydrate metabolism**	10	10	0	0
**Transporter**	8	6	2	0
**Membrane trafficking**	8	5	2	1
**DNA and RNA processing/modification**	30	11	19	0
**Cytoskeleton**	15	4	10	1
**Miscellaneous**	37	12	24	1
**Uncharacterized protein**	39	15	23	1

### DPPs involved in transcription, translation and post-translational modification

Twenty DPPs, associated with transcription factors (TFs) and translation and post-translational modification, were examined under Al^3+^ stress (Table 1, S2 and S9 Tables). For the TFs, Al^3+^ enhanced the phosphorylation of the zinc finger protein GIS2 (Glyma.04G008700.1.p) and MYB183 (Glyma.06G187600.1.p), while it suppressed the phosphorylation of GT-2 (Glyma.06G149900.2.p) and bZIP56 (Glyma.18G283800.2.p). Additionally, 1 transcriptional coactivator (Glyma.06G155900.1.p) was hypophosphorylated under Al^3+^ stress.

For translation, eukaryotic translation initiation factors (EIF) (Glyma.03G106500.1.p, Glyma.08G340500.2.p and Glyma.U037300.1.p) were hypophosphorylated by Al^3+^, while EIF 4B2 was upregulated and downregulated at Ser_510_ and Ser_501_, respectively. Peptidyl-prolyl cis-trans isomerases, which allow proteins to fold into their correct conformations, were hypophosphorylated by Al^3+^, including CYP63 (Glyma.03G157900.2.p) and FKBPs (Glyma.01G231400.1.p, Glyma.11G014400.1.p and Glyma.20G055800.2.p). In addition, Al^3+^ upregulated the phosphorylation of the ribosomal proteins S6 and S11. Regarding post-translational modifications, 4 E3 ubiquitin-protein ligases were found, with ATL6 (Glyma.10G156500.1.p), LIN1 (Glyma.10G194500.2.p) and MARCH7 (Glyma.13G139200.3.p) upregulated, and RBBP6 (Glyma.17G143300.2.p) downregulated.

### DPPs involved in carbohydrate metabolism

**Fig 4:**
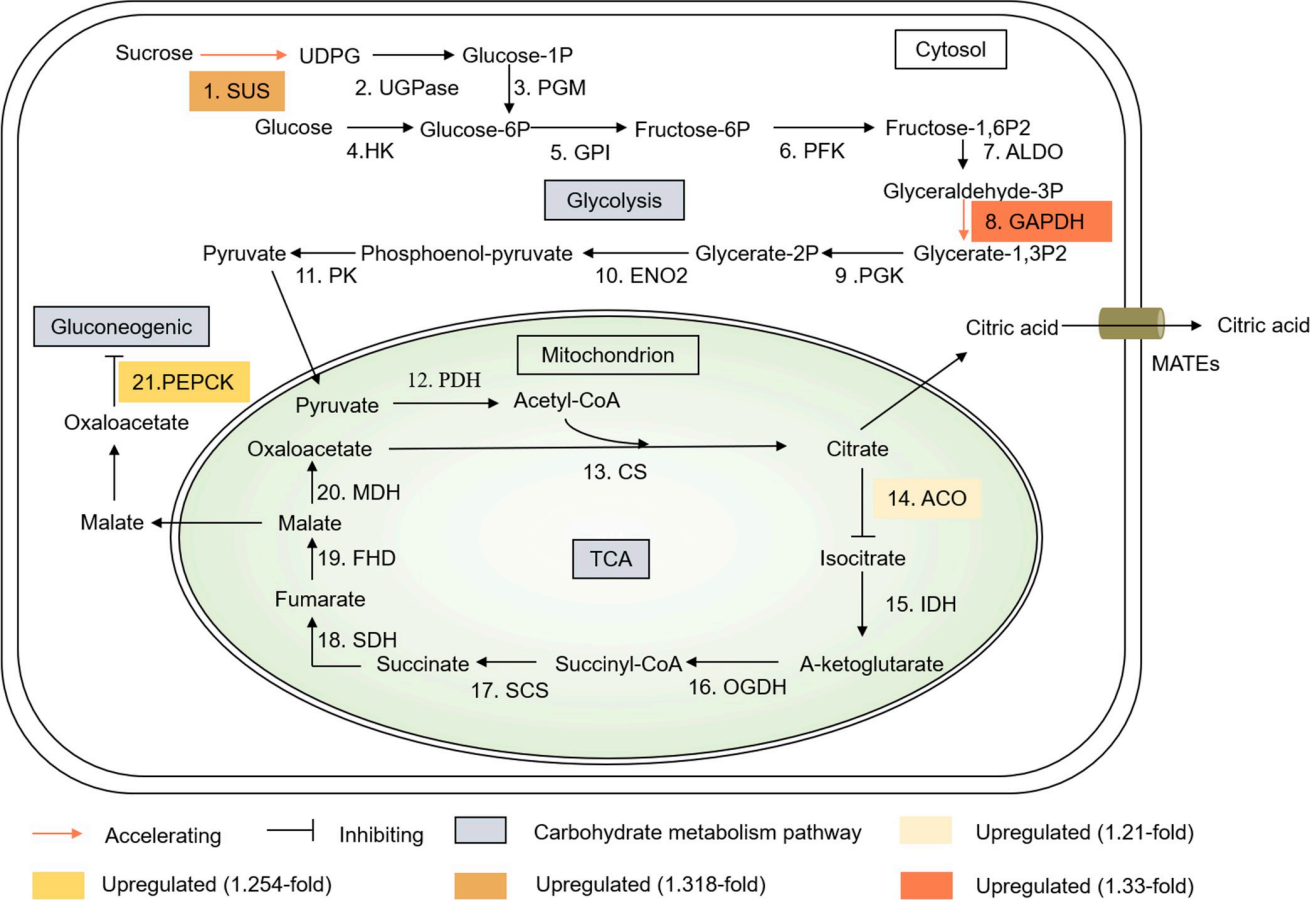
DPPs involved in carbohydrate metabolism. 1. SUS, sucrose synthase; 2. UGPase, UDP-glucose pyrophosphorylase; 3. PGM, phosphoglucomutase; 4. HK, hexokinase; 5. GPI, glucose-6-phosphate isomerase; 6. PFK, phosphofructokinase; 7. ALDO, aldolase; 8. GAPDH, glyceraldehyde-3-phosphate dehydrogenase; 9. PGK, phosphoglycerate kinase; 10. ENO2, enolase2; 11. PK, pyruvate kinase; 12. PDH, pyruvate dehydrogenase; 13. CS, citrate synthase; 14. ACO, aconitate hydratase; 15. IDH, isocitrate dehydrogenase; 16. OGDH, α-ketoglutarate dehydrogenase complex; 17. SCS, succinyl-CoA synthetase; 18. SDH, succinate dehydrogenase; 19. FHD, fumarase; 20. MDH, malate dehydrogenase; 21. PEPCK, phosphoenolpyruvate carboxykinase.

The Al^3+^-induced release of citrate plays an important role in Al^3+^ resistance in soybean [25]. The synthesis of citric acid occurs in the tricarboxylic cycle (TCA), which plays an important role in carbon metabolism. In total, 10 DPPs involved in carbohydrate metabolism were significantly increased in response to Al^3+^ stress (Table 1, S2 and S10 Tables). Among the DPPs involved in the TCA, aconitate hydratase (ACO) (Glyma.01G162800.1.p) was hyperphosphorylated (Fig 4). Regarding glycolysis and gluconeogenesis, phosphorylation of glyceraldehyde-3-phosphate dehydrogenase (GAPDH, Glyma.04G193500.1.p) and phosphoenolpyruvate carboxykinase (PEPCK, Glyma.01G019200.1.p) was observed to be upregulated, respectively (Fig 4). For sucrose metabolism, sucrose synthase (SUS, Glyma.15G182600.2.p) was found to be hyperphosphorylated (Fig 4). In the oxalate cycle, oxalate-CoA ligase (OCL, Glyma.11G198300.1.p) was hyperphosphorylated by more than 2-fold by Al^3+^.

### DPPs involved in transporters

Transporters are critical for the alleviation of Al^3+^-induced root growth inhibition [26], Al^3+^-induced secretion of OAs [27] and Al^3+^ uptake, sequestration and distribution [7]. In total, 8 transporters were identified (Table 1, S2 and S11 Tables). Among them, 6 DPPs were increased, while 2 DPPs were decreased in regard to phosphorylation. The upregulated DPPs belonged to amino acid transporters (2 lysine histidine transporters, Glyma.01G161100.1.p and Glyma.11G082700.1.p), the ABC transporter family (1 ABC transporter, Glyma.13G119000.2.p), inorganic ion transporters (1 potassium transporter, Glyma.19G263100.1.p and 1 high affinity nitrate transporter, Glyma.18G141900.1.p), etc. The downregulated DPPs belonged to amino acid transporters (1 lysine histidine transporter, Glyma.15G068700.2.p) and inorganic ion transporters (1 boron transporter 1, Glyma.03G222300.1.p).

## Discussion

To the best of our knowledge, this study represents one of the first quantitative phosphoproteomic analyses characterizing responses to Al^3+^ in TBS seedlings. The findings showing that the Al^3+^ treatment inhibited root growth, reduced the root citrate content and increased root citrate secretion (Fig 1) make it possible to analyze Al^3+^-response proteins in TBS. We believe that the significant decrease of the citrate content of root tips after the Al^3+^ treatment was related to the enhanced citrate exudation. The error rate and length distribution of the enriched peptides, which are consistent with the properties of tryptic peptides, indicate the accuracy of the mass spectrometry data (S1 Fig). Particularly, the identified phosphorylation sites and DPPs in response to Al^3+^ stress provide a rich source for use in investigating the multiple mechanisms underlying Al^3+^ tolerance in TBS.

### Global analysis of DPPs under Al^3+^ stress

Inhibition of root elongation can be easily observed under Al^3+^ stress [28]. However, TBS root released citrate to relieve toxicity [14]. In our work, most of the DPPs associated with spliceosome and ribosome biogenesis in eukaryotes (Fig 3) were hypophosphorylated, and hyperphosphorylated DPPs of membrane trafficking might contribute to the inhibition of root growth [29–31]. On the other hand, the majority of the DPPs categorized as belonging to signal transduction and carbohydrate metabolism (Fig 3) underwent hyperphosphorylation, which was conducive to Al^3+^-induced signal transduction and citric acid synthesis and release against Al^3+^ toxicity.

### DPPs involved in signal transduction

**Fig 5:**
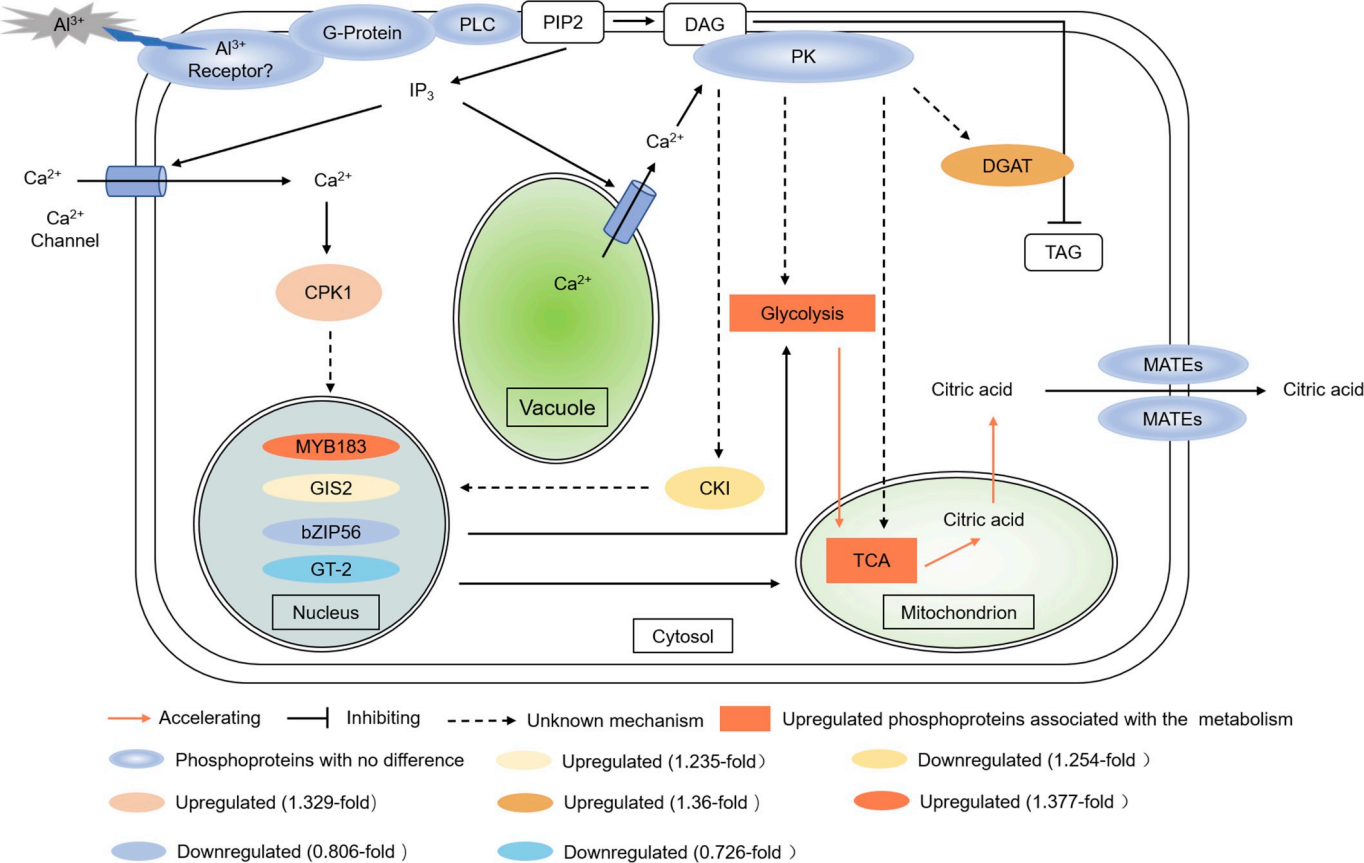
Putative model illustrating the signaling cascades of the Al^3+^-induced exudation of citrate from TBS roots. Phosphoinositide phospholipase C (PLC), phosphatidylinositol 4, 5-phosphate (PIP2), diacylglycerol (DAG), diacylglycerol acyltransferases (DGAT), triacylglycerol (TAG), protein kinases (PK), inositol triphosphate (IP3), calcium-dependent protein kinase (CPK), transcription factor (TF), casein kinase I (CKI), tricarboxylic acid (TCA).

Our recent data established that Al^3+^ sensing in *Stylosanthes* requires the G-protein-mediated signaling pathway, which includes PLC, phosphatidylinositol 4, 5-phosphate (PIP2), IP3, DAG, Ca^2+^ and protein kinases [9]. Proteins associated with the G-protein-mediated signaling pathway, DGAT and kinase cascades (CPK1 and CKI) were detected in the present study (S1, S2 and S8 Tables). The activity of DGAT was inhibited by phosphorylation [32]. Therefore, hyperphosphorylation of DGAT promoted signal transduction via DAG accumulation under Al^3+^ stress. Furthermore, phosphorylation of CPK1 and CKI was enhanced by the Al^3+^ treatment (Fig 5, S2 and S8 Tables). Actually, CPKs have been reported to phosphorylate target proteins, such as transcription factors (TFs), to specify the reprogramming of genes [33]. SeCKI has been reported to regulate *SeFAD2* expression via phosphorylation of the SebHLH transcription factor [34]. Taken together, the results show that the Al^3+^ tolerance of soybean involves phosphorylation in G-protein-mediated Al^3+^ signaling and kinase cascades.

### DPPs involved in transcription

Evidence shows that transcription factors such as the MYB [16], WRKY [35], zinc finger protein [27], MADS-box [36], NAC [37, 38] and bZIP [39] families are required for Al^3+^ responses. In our work, the zinc finger protein GIS2 and MYB183 were hyperphosphorylated under Al^3+^ stress, while bZIP56 and GT-2 were hypophosphorylated (Fig 5, S2 and S9 Tables). Soybean tolerance to salinity was enhanced by inoculation with rhizobia via inhibiting the phosphorylation of GmMYB183 and inhibiting its activity in *GmCYP81E11* expression transcriptional regulation [40]. However, GmMYB183 was hyperphosphorylated at Ser_36_ in response to Al^3+^ stress. This might be due to GmMYB183 being phosphorylated by different kinases in response to different stimuli. In *Arabidopsis*, the phosphorylation of MYB41 and the zinc finger transcriptional regulator ZAT6 were required for the tolerance of salt during seed germination and root growth [41, 42]. Phosphorylated MYB15 promotes *CBF3* expression, which regulates root growth under freezing stress, via reducing its binding to the *CBF3* promoter [43, 44]. The phosphorylation of AREB1, which is a bZIP, is required for the expression of ABA-inducible genes [45]. STOP1, which is a Cys2/His2 type zinc-finger protein, regulates the expression of Al^3+^-resistance genes in *Arabidopsis*, and the phosphorylation of STOP1 enhances malate exudation under Al^3+^ stress [46]. Therefore, we speculated that the TFs, detected in our work, regulated the expression of genes associated with root growth and citric acid synthesis based on phosphorylation or dephosphorylation.

### DPPs promotes citric acid synthesis under Al^3+^ stress

It has been recognized that Al^3+^ tolerance in beans relies on their capacity for citric acid synthesis, which is related to carbohydrate metabolism [24]. The DPPs, associated with carbohydrate metabolism, were all hyperphosphorylated in this work (S2 and S10 Tables). In higher plants, sucrose, as the main form of carbohydrate transport, can be digested into glucose, contributing to glycolysis. SUS, which is activated by phosphorylation [47], converts sucrose into glucose-6P, which plays a role in the glycolytic pathway. In the present study, hyperphosphorylated SUS promoted the glycolytic pathway under Al^3+^ stress (Fig 4). Meanwhile, GAPDH, which is an enzyme involved in glycolysis, was shown to be hyperphosphorylated after the Al^3+^ treatment (Fig 4). The enzyme was activated by phosphorylation [48], and then, Al^3+^ stress accelerated the glycolytic rate.

Citric acid metabolism, as part of the tricarboxylic acid (TCA) cycle, occurs in mitochondria. Our data revealed that ACO was hyperphosphorylated in response to Al^3+^ stress (Fig 4). ACO, which is an enzyme that catalyzes the transformation of citric acid into isocitrate in the TCA cycle, was downregulated by exogenous SA under Al^3+^ stress enhancing citrate release from soybean roots [49]. ACO was detected to be hyperphosphorylated in TBS roots under Al^3+^ stress, and we speculated that the phosphorylation of ACO weakened its activity and increased the accumulation of citric acid.

In addition, hyperphosphorylated PEPCK was found in our study (Fig 4). It has been reported that PEPCK activation is suppressed by phosphorylation [50, 51]. Hence, the activation-suppressed PEPCK results in an accumulation of oxaloacetate, contributing to citric acid synthesis. Overall, the phosphorylation of proteins linking carbohydrate metabolism pathways facilitates citric acid synthesis for Al^3+^ detoxification in TBS roots.

## Conclusions

This study explored the mechanism of Al^3+^ resistance through global phosphorylation levels in TBS plants. According to the results, 189 of 1280 quantified proteins were significantly differentially phosphorylated in response to Al^3+^ stress. Among them, 88 DPPs were upregulated, 96 DPPs were downregulated, and 5 DPPs were both up-/downregulated in regard to phosphorylation. Functional analysis of DPPs, together with PPI analysis, revealed DPPs associated with the inhibition of root growth and citric acid synthesis metabolism. Of these, the DPPs of the Al^3+^ signaling cascades comprised CPK1, CKⅠ and DGAT, and those of the citric acid synthesis metabolism included SUS, GAPDH, ACO and PEPCK. As a consequence, our work provides important data for understanding the Al^3+^ signaling and enhanced citric acid synthesis metabolism in response to Al^3+^ stress in TBS plants.

## Supporting information

S1 Fig Quality control validation of mass spectrometry data. **(A)** Volcano map of the error rate distribution for mass spectrometry; **(B)** Length distribution of the phosphorylated peptides.
(TIF)

S2 Fig GO function classifications of differentially phosphorylated proteins (DPPs) in Al^3+^-treated plants compared with untreated controls. **(A)** Biological process; **(B)** Cellular component; **(C)** Molecular function.
(TIF)

S3 Fig Subcellular localization prediction of DPPs in Al^3+^-treated plants compared with untreated controls. **(A)** All DPPs; **(B)** Upregulated DPPs; **(C)** Downregulated DPPs.
(TIF)

S4 Fig Gene ontology (GO) enrichment analysis of DPPs according to cellular components, molecular functions and biological processes. (TIF)

S5 Fig KEGG pathway enrichment analysis of the 50 μM versus 0 μM Al^3+^ treatments. Enriched KEGG pathways for upregulated proteins **(A)** and downregulated proteins **(B)**.
(TIF)

S1 Table The detail information of all identified peptides. (XLSX)

S2 Table The detail information of differential phosphorylated proteins and phosphosites. (XLSX)

S3 Table GO enrichment of up- and downregulated phosphoproteins. (XLSX)

S4 Table KEGG pathway of differential phosphoproteins. (XLSX)

S5 Table Phosphorylation motifs of all sites in proteins of *Glycine max* cv. Tamba. (XLSX)

S6 Table Summary of phosphoproteins affected by Al^3+^. (XLSX)

S7 Table Node Information of PPI. (XLSX)

S8 Table DPPs associated with signal transduction. (XLSX)

S9 Table DPPs associated with transcription, translation and posttranslational modification. (XLSX)

S10 Table DPPs associated with carbohydrate metabolism. (XLSX)

S11 Table DPPs associated with transporters. (XLSX)
