# Supplementary figures and images for: Quantitative phosphoproteomic analysis provides insights into the aluminum-responsiveness of Tamba black soybean
Source: PLoS One. 2020 Aug 19;15(8):e0237845. doi: 10.1371/journal.pone.0237845 (PMC7437914; doi:10.1371/journal.pone.0237845)

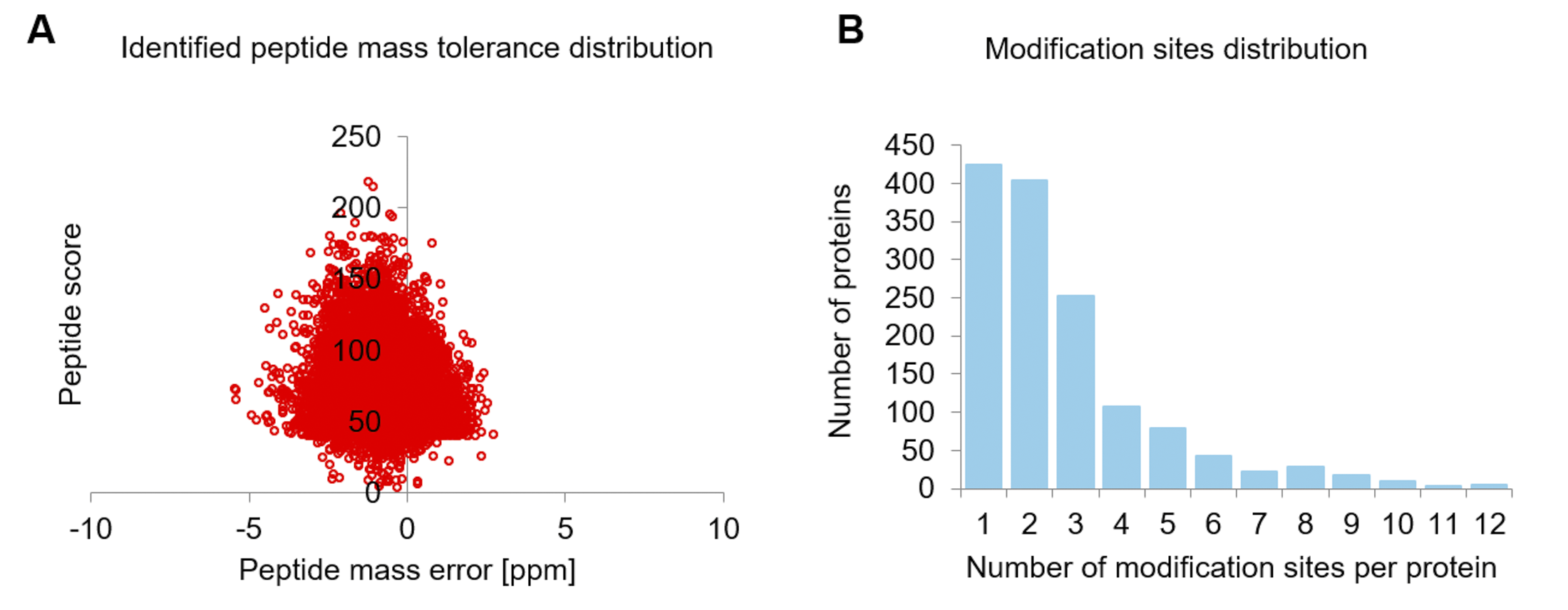

Supplement: S1 Fig — (A) Volcano map of the error rate distribution for mass spectrometry; (B) Length distribution of the phosphorylated peptides. (TIF) [file pone.0237845.s001.tif]

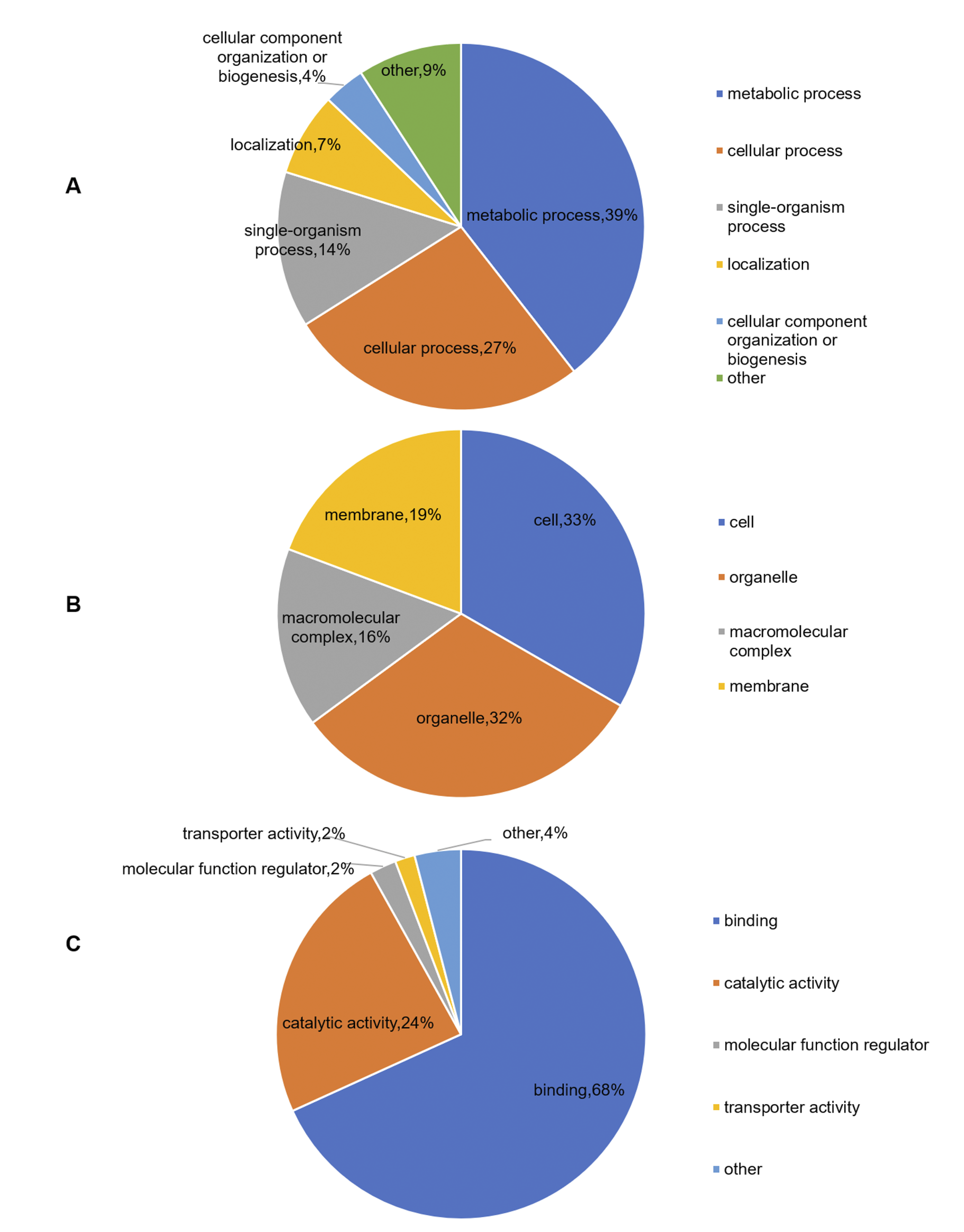

Supplement: S2 Fig — (A) Biological process; (B) Cellular component; (C) Molecular function. (TIF) [file pone.0237845.s002.tif]

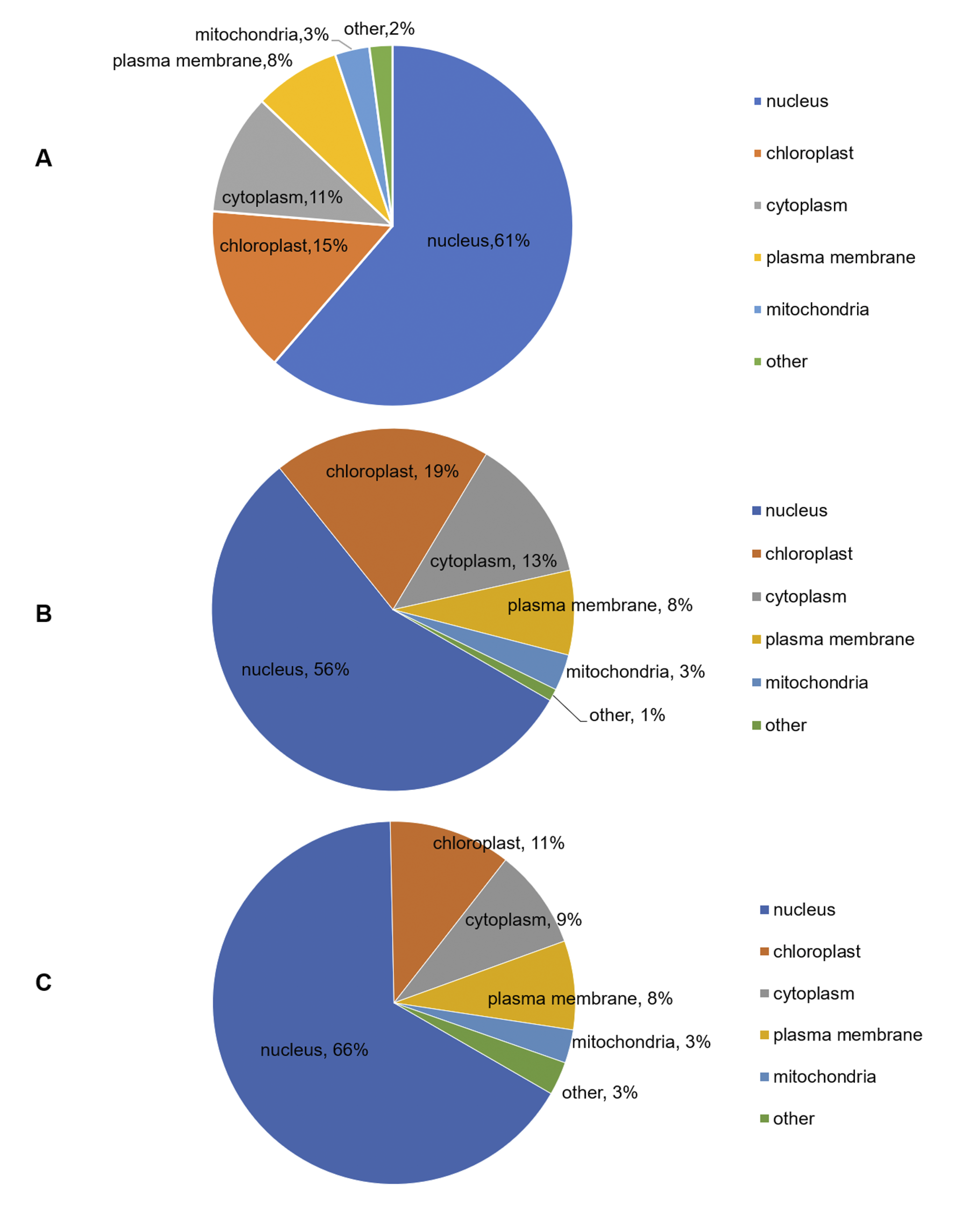

Supplement: S3 Fig — (A) All DPPs; (B) Upregulated DPPs; (C) Downregulated DPPs. (TIF) [file pone.0237845.s003.tif]

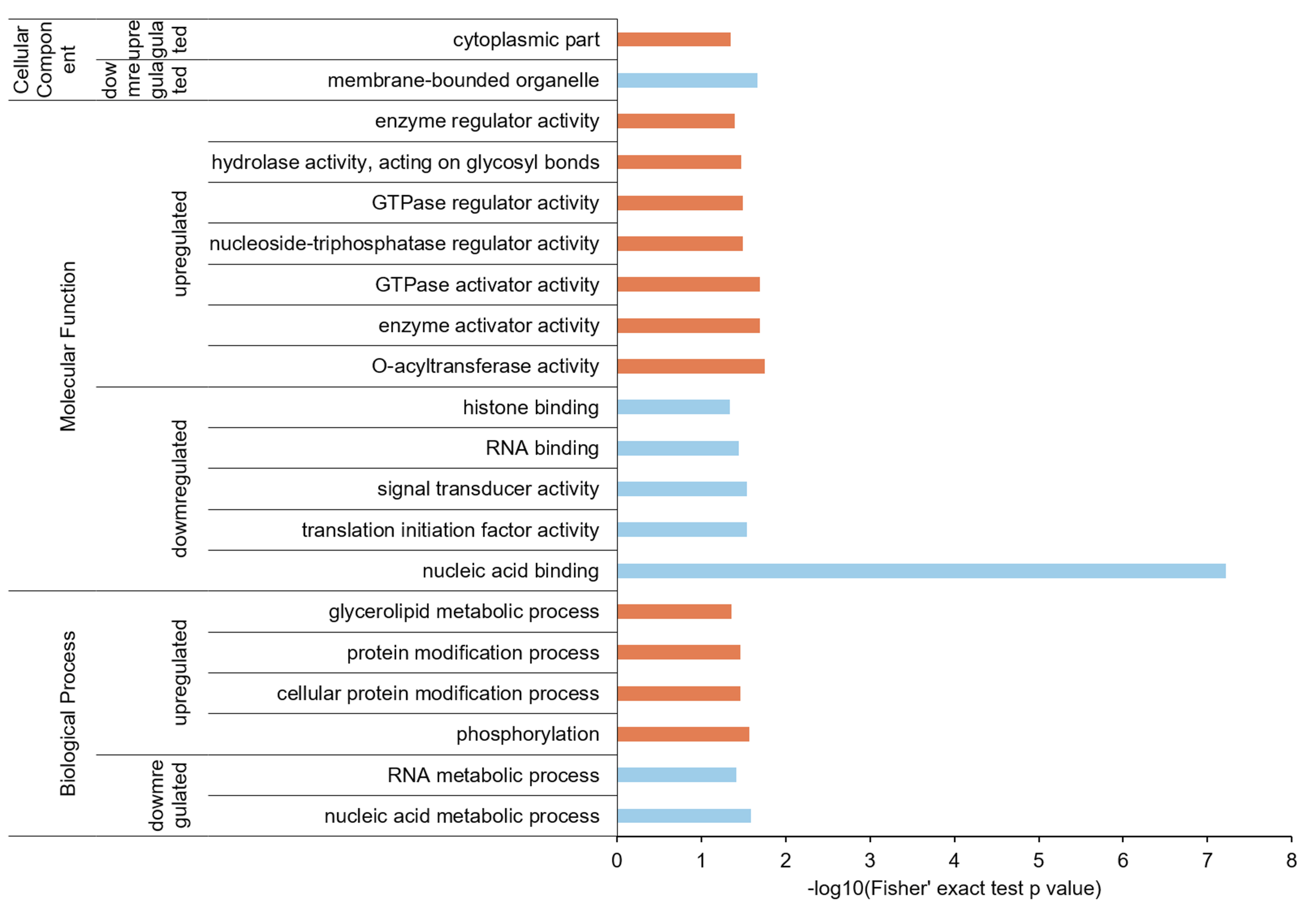

Supplement: S4 Fig — (TIF) [file pone.0237845.s004.tif]

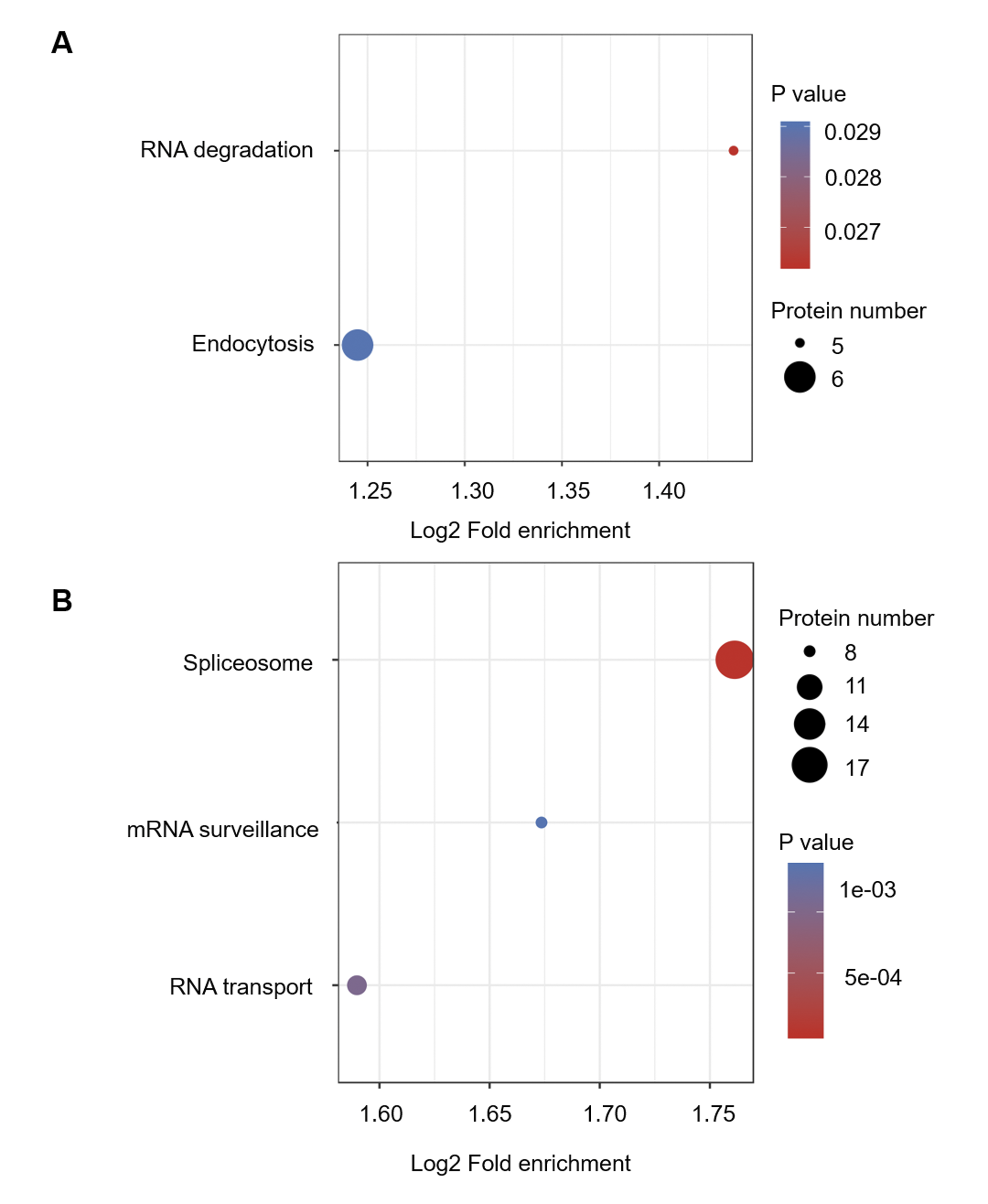

Supplement: S5 Fig — Enriched KEGG pathways for upregulated proteins (A) and downregulated proteins (B). (TIF) [file pone.0237845.s005.tif]
